# Carbon Emission Accounting and the Carbon Neutralization Model for a Typical Wastewater Treatment Plant in China

**DOI:** 10.3390/ijerph20010140

**Published:** 2022-12-22

**Authors:** Chenxi Pang, Xi Luo, Bing Rong, Xuebiao Nie, Zhengyu Jin, Xue Xia

**Affiliations:** 1 College of Life and Environmental Sciences, Minzu University of China, Beijing 100081, China; 2 Yangtze Ecology and Environment Co., Ltd., Wuhan 430062, China; 3 Beijing Urban Construction Design & Development Group Co., Limited, Beijing 100037, China; 4 Beijing Enterprises Water Group (China) Investment Limited, Beijing 100102, China

**Keywords:** wastewater treatment plant, carbon neutrality, energy self-sufficiency, carbon emission reduction, Gaobeidian

## Abstract

To reduce carbon emissions and achieve carbon neutrality in China, it is pivotal to explore low-carbon wastewater treatment processes and carbon-neutral wastewater treatment plants (WWTPs). This study investigated the Beijing Gaobeidian WWTP to explore the current energy consumption and carbon emission status of representative WWTPs in China. Furthermore, it explored a possible low-carbon operating model. Results show that the current total energy consumption of Gaobeidian WWTP is 280,717 MWh/y, while its energy recovery is 268,788 MWh/y. As a result, the energy neutralization ratio is 95.8%, and the plant is close to reaching energy neutrality. The carbon emission of this plant is 446,468 t/y. However, it reduced its carbon emissions by 252,994 t/y and reached only 56.7% of carbon neutrality. Although the plant almost reached energy neutrality, it has a long way to go before reaching carbon neutrality. It was found that a subsequent increase in the recovery of residual heat from secondary effluent can increase the energy and carbon neutralization ratio to 523.1% and 219.0%, respectively, meaning that the WWTP can become a power production unit and a carbon sink. This study can provide a reference for exploring efficient energy use and reaching carbon neutrality for domestic WWTPs.

## 1. Introduction

In the context of improved sewage treatment technology and severe climate change, the energy consumption of wastewater treatment plants (WWTPs) in China has become an important topic. Currently, the energy consumption of WWTPs has accounted for more than 2% of the total energy consumption in China [1,2]. In addition, scientists and academics are devoting more attention to carbon emissions from WWTPs [3]. According to statistics, China’s carbon emission from WWTPs is around 187,000 t (CO_2_)/d, accounting for approximately 2~5% of the total carbon emissions of the whole country [4]. Therefore, it is imperative to reduce both energy consumption and carbon emission in the wastewater treatment industry. Given the low cost of wastewater treatment and its remarkable effect on carbon emission reduction, some developed countries have incorporated wastewater treatment into their carbon emission reduction plans [5,6,7,8]. By optimizing the anaerobic ammonia oxidation process, the Austrian Strass WWTP reached carbon neutrality and 100% energy self-sufficiency. Finland’s Kakolanmäki WWTP attaches importance to the recovery of energy from the effluent so that it recovers energy and operates in a carbon-neutral manner while meeting effluent quality standards [9]. Therefore, attention needs to be directed toward reducing greenhouse gas (GHG) emissions from WWTPs.

GHG emissions from WWTPs are divided into direct and indirect ones. Direct emissions are CO_2_, N_2_O, and CH_4_, directly emitted during the wastewater and sludge treatment process, like CO_2_ released as a result of the degradation of organic matter during wastewater treatment, often called biogenic carbon, and CO_2_ from decomposed organic matter contained in petrochemical products, called fossil carbon. Although biogenic carbon is an important component, the Intergovernmental Panel on Climate Change (IPCC) does not include it in GHG emission calculations, given that these are of biogenic origin and not caused by the application of WWTPs [10,11,12]. Indirect emissions represent emissions resulting from the consumption of electricity and chemicals in WWTPs [13]. Electricity is a major component of indirect GHG emissions from WWTPs, and over 99% of it is consumed by aeration equipment, pumps, mixing motors, and other facilities [14,15,16]. Compared to carbon emissions from electricity consumption, carbon emissions from chemical dosing are relatively small [12].

To reduce carbon emissions from WWTPs, researchers proposed several measures for carbon emission reduction. For instance, modifying equipment such as blower aeration systems and optimizing the chemical dosing process are sustainable ways of reducing energy consumption and carbon emissions. In order to increase carbon sinks and achieve low-carbon operation, the chemical energy contained in the sewage needs to be used and converted into a stable energy carrier [17]. This is mainly carried out through sludge treatment, especially through the recovery of electrical and thermal energy from biogas cogeneration [18,19]. Using treated sludge in land application instead of a fertilizer also plays a role in carbon emission reduction. In terms of potential development, carbon emission reduction can also be achieved through the utilization of waste heat from secondary effluent, the development of solar or wind energy for power generation, and electricity recovery from wastewater using bioelectrochemical technologies [12,20,21]. In addition, organic and nitrogen containing pollutants can be largely removed from wastewater by advanced treatment, thus reducing the total amount of pollutants discharged into natural water bodies. Compared with direct discharge of wastewater, this can reduce the amount of GHGs released into the atmosphere since incomplete degradation of pollutants in natural water bodies will produce GHGs like CH_4_ and N_2_O. Therefore, water quality improvement has a positive effect on carbon emission reduction.

As a typical secondary WWTP in Beijing, Gaobeidian WWTP made a breakthrough in energy saving and power production through advanced anaerobic digestion technology. This study focused on the current state of Gaobeidian WWTP and investigated whether the plant can be energy neutral by calculating both the energy consumption and energy recovery of the WWTP. On the basis of energy calculations, direct and indirect carbon emissions during the operation of the WWTP were calculated. The carbon emission reductions corresponding to water quality improvement, sludge treatment, and residual heat recovery were also calculated and summarized. The values of carbon emission and carbon emission reduction were then compared to evaluate the total net carbon emission of the WWTP and analyze whether it can achieve carbon neutrality. The model for optimizing carbon neutrality was discussed throughout the process of carbon accounting and a new model of energy saving and carbon emission reduction was proposed to promote carbon neutrality.

## 2. Materials and Methods

### 2.1. The Definition of the Carbon Footprint

**Figure 1:**
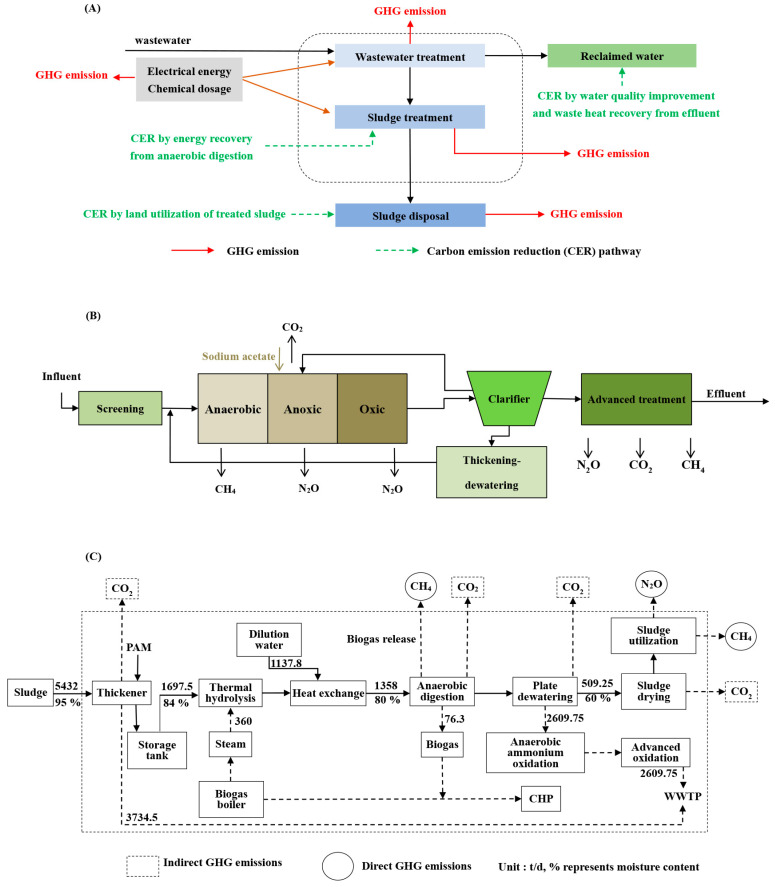
The system boundaries of (**A**) Gaobeidian WWTP, (**B**) the wastewater treatment process, and (**C**) the sludge treatment process for calculating the carbon footprint.

The carbon footprint in this study is defined as the direct and indirect GHG emissions caused by wastewater and sludge treatment within defined system boundaries. The GHG emissions accounted for include carbon dioxide (CO_2_), methane (CH_4_), and nitrous oxide (N_2_O). All are converted into carbon dioxide equivalents (CO_2_) based on 100-year global warming potentials (GWPs), namely, 1 for CO_2_, 21 for CH_4_, and 298 for N_2_O [5]. Because most of the organic matter in wastewater is biogenic and generally not accounted for in carbon emission inventories and because the proportion of fossil carbon in wastewater can be overlooked [20], this study only focused on CO_2_ emission due to external chemical input. As shown in Figure 1, the calculation of direct GHG emissions include direct emissions from the wastewater treatment process (e.g., CO_2_ emissions from an external carbon source, N_2_O emissions from the nitrification/denitrification process, and CH_4_ emissions from the anaerobic zone of A^2^O process), the sludge treatment process (e.g., CH_4_ emissions from anaerobic digestion), and the sludge disposal process (e.g., CH_4_ and N_2_O emissions from land application of sludge). The calculation of indirect GHG emissions included emissions from electricity and chemical consumptions within the defined system boundary [22].

### 2.2. The Current State of Gaobeidian WWTP

Gaobeidian WWTP is the largest secondary WWTP in Beijing, with a treatment scale of 100 × 10^4^ m^3^/d. At present, a modified version of the A^2^O process is used for wastewater treatment. The secondary effluent is further treated using the denitrification biofilter, ultrafiltration, ozone contact reactor, and ultraviolet disinfection processes. The quality of the effluent from this WWTP meets the Grade B standard of the Beijing local Discharge Standard of Water Pollutants for Municipal Wastewater Treatment Plants (DB11/890-2012) [23]. The average influent and effluent water quality indicators of the Gaobeidian WWTP in 2020 are shown in Table 1. The sludge system adopts the main processes of thermal hydrolysis, advanced anaerobic digestion, plate and frame dewatering, and land application of treated sludge.

### 2.3. Estimation of Direct GHG Emissions

#### 2.3.1. Direct GHG Emissions from the Wastewater Treatment Process

Sodium acetate is currently used as an additional carbon source in Gaobeidian WWTP to promote nitrate and nitrite reduction by denitrifying bacteria. The carbon source itself was oxidized to CO_2_. The direct CO_2_ emissions at this stage were calculated using Equation (1) as follows:

(1) $$m_{CO_{2},\mathrm{carbon}\;\mathrm{source}}=\frac{n\times M_{CO_{2}}}{M_{NaAc}}\times m_{\mathrm{carbon}\;\mathrm{source}}$$

where $m_{CO_{2},\mathrm{carbon}\;\mathrm{source}}$ is the CO_2_ emission that resulted from the addition of sodium acetate (t/y), $m_{\mathrm{carbon}\;\mathrm{source}}$ is the amount of sodium acetate (t/y), *n* is the amount of CO_2_ produced per mol of oxidized sodium acetate (*n* = 2), and $M_{CO_{2}}$ and $M_{NaAc}$ are the relative molecular masses of CO_2_ and sodium acetate, respectively ($M_{CO_{2}}=44\;g/\mathrm{mol},\;M_{NaAc}=82\;g/\mathrm{mol})$.

N_2_O emissions mainly come from the intermediates of nitrification and denitrification processes. IPCC uses an empirical method to estimate the N_2_O emission coefficient of WWTPs. The N_2_O emission coefficient ranges from 0.008 to 0.39 kg N_2_O/kg TN for WWTPs based on the aerobic process [9]. In this study, an empirical coefficient of 0.035 kg N_2_O/kg TN was used for traditional nitrification-denitrification techniques in reference to previous studies [25]. The N_2_O emission from the sewage treatment process was calculated using Equation (2) as follows:

(2) $$m_{N_{2}O,\mathrm{st}}=Q\times TN_{\inf}-TN_{\mathrm{eff}}\times EF_{N_{2}O,\mathrm{st}\;}$$

where $m_{N_{2}O,\mathrm{st}}$ is the annual discharge of N_2_O in the sewage treatment process (t/y), *Q* is the treated sewage volume (m^3^/y), $TN_{\inf}$ and $TN_{\mathrm{eff}}$ are the total nitrogen (TN) concentrations in influent and effluent, respectively (mg/L), and $EF_{N_{2}O,\mathrm{st}}$ is the empirical coefficient of the discharge of N_2_O (0.035 kg N_2_O/kg TN).

CH_4_ emission from the anaerobic zone of the A^2^O process can be calculated using Equation (3) as follows:

(3) $$m_{CH_{4},\mathrm{st}}=m_{COD}\times EF_{CH_{4},\mathrm{st}}-R$$

where $m_{CH_{4},\mathrm{st}}$ is the amount of CH_4_ released from the anaerobic process during sewage treatment (t/y), $m_{COD}$ is the total amount of COD in the influent (t/y; *m_COD_* = *Q* × *COD_inf_*, *COD_inf_* is the influent concentration of COD), $EF_{CH_{4},\mathrm{st}}$ is the CH_4_ emission coefficient during the sewage treatment process (0.025 kg CH_4_/kg of COD as recommended by previous studies) [26], and *R* is the amount of recovered CH_4_ (it has a 0 value in this study since it is difficult to recover methane during wastewater treatment).

#### 2.3.2. Direct GHG Emissions from the Sludge Treatment and Disposal Process

During the sludge treatment process, approximately 5% of the biogas leaks from pipes and escape into the atmosphere, resulting in direct carbon emissions [27]. This carbon emission can be calculated using Equation (4) as follows:

(4) $$m_{ad,CH_{4}}=\eta \times Q_{\mathrm{biogas}}\times 65\%\times \frac{M_{CH_{4}}}{V_{m}}=2.127\times 10^{-5}\times Q_{\mathrm{biogas}}$$

where $m_{ad,CH_{4}}$ is the amount of CH_4_ released into the atmosphere during anaerobic digestion of sludge (t/y), η is the uncontrollable methane leakage ratio during biogas production (5%), $Q_{\mathrm{biogas}}$ is the amount of biogas produced by anaerobic digestion of sludge (m^3^/y, which is 2.555 × 10^7^ m^3^/y for Gaobeidian WWTP [28]), 65% is the volume fraction of methane in biogas [20], $M_{CH_{4}}$ is the relative molecular mass of CH_4_ (16 g/mol), and *V_m_* is the molar volume of gas (24.451 L/mol at 25 °C and one atmospheric pressure).

The treated sludge from Gaobeidian WWTP Is currently used in forest land application, horticulture and nurseries, and sandy wasteland improvement. GHGs such as CH_4_ and N_2_O are produced during land application of treated sludge. The amount of GHGs released from this process can be calculated using Equations (5) and (6) as follows:

(5) $$m_{land,N_{2}O}=0.011\times W_{land}\times \omega_{N}\times \frac{M_{N_{2}O}}{n^{,}\times M_{N}}$$

(6) $$m_{land,CH_{4}}=0.003\cdot W_{land}$$

where $M_{land,N_{2}O}$ and $M_{land,CH_{4}}$ are the amount of N_2_O and CH_4_ released from land application of treated sludge (t/y), respectively, $W_{land}$ is the volume of dry sludge used in land application (t/y), $\omega_{N}$ is the mass fraction of N in dry sludge (0.12 [29]), $M_{N_{2}O}$ and *M_N_* are the molecular masses of N_2_O and N, respectively ($M_{N_{2}O}=44\;g/\mathrm{mol},\;M_{N}=14\;g/\mathrm{mol}$), *n’* is the amount of N needed per mole of the produced N_2_O (*n’* = 2), and 0.011 and 0.003 are the emission factors of N_2_O and CH_4_ from land application of treated sludge [29], respectively.

### 2.4. Calculation of Indirect GHG Emissions

Indirect carbon emissions from WWTPs are byproducts of electricity, heat, and chemical consumption. Electrical energy is consumed during the processes of aeration, sludge transportation, sludge dewatering, etc. Moreover, heat is consumed during sludge pyrolysis, while chemicals are consumed through dosing of additional carbon sources, phosphorus removal chemicals, and disinfectants.

Carbon emissions from electricity, heat, and chemical consumption can be calculated using Equations (7)–(9), respectively.

(7) $$m_{ec}=EF_{ec}\cdot \;C_{ec}$$

(8) $$m_{hc}=EF_{hc}\cdot \;C_{hc}$$

(9) $$m_{cc}=\sum_{i=1}^{N}C_{cc,i}EF_{cc,i}$$

where $m_{ec}$, $m_{hc}$, and $m_{cc}$ are indirect carbon emissions from electricity, heat, and chemical consumption, respectively (t/y, carbon emissions are calculated in CO_2_ equivalents), $C_{ec}$ is electricity consumption (MWh/y), $C_{hc}$ is heat consumption (GJ/y), $C_{cc,i}$ is the annual consumption of agent *i* (t/y), $EF_{ec}$ is the CO_2_ emission factor of electricity consumption (0.604 t/MWh for the Beijing municipal grid [30]), $EF_{hc}$ is the emission factor of heat consumption (0.11 t/GJ for the heating system in Beijing [30]), and $EF_{cc,i}$ is the corresponding CO_2_ emission factor of agent *i* (t/t).

### 2.5. Calculation of the Carbon Emission Reduction

#### 2.5.1. Carbon Emission Reduction Resulting from Water Quality Improvement

Carbon emission reduction resulting from water quality improvement can be expressed through the emission factors of the receiving water bodies. It is calculated using Equation (10) as follows:

(10) $$J_{wq}=Q\times \left[21\times BOD_{5,\;inf}-BOD_{5,\;eff}\times 0.06+298\times TN_{inf}-TN_{eff}\times 0.008\right]$$

where $J_{wq}$ is the carbon emission reduction resulting from water quality improvement (t/y), 21 is the CO_2_ emission coefficient equivalent to CH_4_, 298 is the CO_2_ emission coefficient equivalent to N_2_O, *Q* is the effluent volume of the WWTP (m^3^/y), *BOD_5,inf_, BOD_5,eff_, TN_inf_*, and *TN_eff_* are the influent and effluent concentrations of BOD_5_ and TN, respectively (mg/L), 0.06 kg CH_4_/kg BOD_5_ is the CH_4_ emission factor of surface water, and 0.008 kg N_2_O/kg TN is the N_2_O emission factor of surface water [29].

#### 2.5.2. Carbon Emission Reduction Resulting from Sludge Treatment and Disposal

Carbon emission reduction from sludge treatment is achieved by recovering heat and electrical energy from anaerobic digestion. A subsequent treatment of the liquid from sludge digestion also saves energy. Besides, carbon emission reduction from sludge disposal is achieved by using the treated sludge in land application instead of a fertilizer. During land application of sludge, plants absorb 61% of N and 70% of P from sludge. Therefore, sludge offers a sustainable alternative to traditional fertilizers and increases carbon sequestration. Energy consumption during the production of the traditional nitrogen fertilizer, ammonium nitrate (NH_4_NO_3_), is 1 GJ/t, while that during the production of the traditional phosphate fertilizer, calcium superphosphate Ca(H_2_PO_4_)_2_, is 1.3 GJ/t [31]. Hence, the carbon sink resulting from land application of sludge can be calculated according to Equation (11) as follows:

(11) $$S_{land}=W_{land}\cdot 0.604\cdot \left(61\%\times \frac{M_{NH_{4}NO_{3}}}{M_{N}}\times 1\times \omega_{N}+70\%\times \frac{M_{Ca{\left(H_{2}PO_{4}\right)}_{2}}}{M_{P}}\times 1.3\times \omega_{P}\right)$$

where $S_{land}$ is the carbon sink resulting from land application of treated sludge (t/y), $W_{land}$ is the amount of dry sludge in land application (t/y), 0.604 t CO_2_/MWh is the emission factor of electricity consumption of the Beijing municipal grid [30], $M_{NH_{4}NO_{3}}$, $M_{Ca{\left(H_{2}PO_{4}\right)}_{2}}$, and *M_P_* are the relative molecular masses of NH_4_NO_3_, Ca(H_2_PO_4_)_2_, and P ($M_{NH_{4}NO_{3}}=80\;g/\mathrm{mol}$, $M_{Ca{\left(H_{2}PO_{4}\right)}_{2}}=234\;g/\mathrm{mol}$, *M_P_* = 31 g/mol), respectively, and $\omega_{N}$ and $\omega_{P}$ are the mass fractions of N and P in dry sludge (*ω_N_* = 0.12, *ω_P_* = 0.02), respectively.

#### 2.5.3. Carbon Emission Reduction Resulting from the Recovery of Residual Thermal Energy

To estimate the potential of reducing carbon emission by heat recovery, a thermal energy recovery model developed by Hao et al. was used for Gaobeidian WWTP [32]. The model calculates the energy that can be extracted from the secondary effluent.

The theoretical heating or cooling capacity contained in the effluent can be calculated using Equation (12) as follows:

(12) A=M×ΔT×C

where *A* denotes the heating or cooling capacity contained in the secondary effluent (kJ), *M* denotes the mass of the secondary effluent (kg), ΔT denotes the temperature difference between the influent and effluent when using the secondary effluent for cooling or heating (°C), and *C* denotes the specific heat capacity of the secondary effluent (4.18 kJ/(kg·°C)).

A water-source heat pump system can be used to extract thermal energy from the secondary effluent. Therefore, the heating or cooling capacity released from this system can be calculated using Equation (13) [9] as follows:

(13) $$A_{H∕C}=A\pm W=A\pm \frac{A}{COP\mp 1}$$

where $A_{H∕C}$ is the heating or cooling capacity extracted from the secondary effluent and released from the water-source heat pump (kJ, subscripts H and C represent the heating and cooling operation mode, respectively), *W* is energy consumed by the water-source heat pump, and COP stands for the ratio of heating or cooling capacity to the electricity consumed by the water-source heat pump.

## 3. Results and Discussion

### 3.1. Analysis of the Energy Balance of Gaobeidian WWTP

The energy in Gaobeidian WWTP is mainly consumed by electricity and heating systems (Table 2). The total annual energy consumption of Gaobeidian WWTP was 280,717 MWh/y.

The primary energy recovery pathways of Gaobeidian WWTP include the production of methane from anaerobic digestion for combined heat and power (CHP) generation, heat recovery from the secondary effluent through the water-source heat pump system, and heat extraction from the cooling water of pumping station, air compressors, and ventilation pipes. The sludge treatment and disposal processes of Gaobeidian WWTP and other relevant data are shown in Figure 1C. The total amount of primary and excess sludge was 5432 t/d with a water content of 95% [12]. In the sludge treatment center, the mixed sludge was pumped into a thickener and then dewatered with the addition of polyacrylamide (PAM). The dewatered sludge was transferred to a sludge storage tank where it was thermally hydrolyzed and anaerobically digested for heat and power cogeneration. After the thickening treatment, thermal hydrolysis, and anaerobic digestion, 509 t/d of sludge with a water content of 60% was placed into the plate and frame filter press for further treatment [16]. The biogas production of Gaobeidian WWTP was 2555 × 10^4^ m^3^/y, of which 1285 × 10^4^ m^3^/y was used for heat and power cogeneration, with a cumulative annual power generation of up to 43,040 MWh [28]. The other half of the biogas was used in the boiler system to produce steam for heating the digestion tanks in the thermal hydrolysis stage and heating the WWTP and its surrounding area. The annual energy recovery capacity of the sludge treatment process (i.e., the sum of electrical energy from heat and power cogeneration, heat recovery from flue gas, heat recovery from water cylinder jackets, and steam from boilers) was 178,940 MWh/y, of which 104,920 MWh/y was used for the thermal hydrolysis treatment of sludge, while 20,800 MWh/y [28] was used as an electricity supply for equipment and sludge transportation. Energy generated from anaerobic digestion reduced the energy required for sludge treatment, so surplus energy was used as an output for other processes and treatments.

Heat from the effluent was recovered by a water-source heat pump system located within the plant. This system provided heating or cooling for the plant area by extracting the temperature difference between the effluent and influent temperature. The potential of thermal energy recovery from the secondary effluent was estimated using the thermal energy recovery model [9]. In particular, the amount of secondary effluent that can be used for heat recovery was 339 × 10^6^ m^3^/y [29], while the annual average extractable temperature difference was ~4 °C. Therefore, the theoretical heating or cooling capacity of the secondary effluent (*A*) was 5,668,080 GJ/y. Furthermore, the heating or cooling capacity that could be released from the water-source heat pump system was calculated and shown in Table 3. The heating capacity released from the water-source heat pump system (*A_H_*) was 7,417,487 GJ/y, while the cooling capacity (*A_C_*) was 4,569,615 GJ/y. These are equal to 2,060,413 MWh/y and 1,269,337 MWh/y of electric energy, respectively. After subtracting the electricity consumption of the water-source heat pump system, the net energy production capacity from the heat of the secondary effluent was 1,574,467 MWh/y for heating and 964,208 MWh/y for cooling. Assuming that heating and cooling each last for 6 months per year, a theoretical annual energy recovery of 1,269,337 MWh can be obtained from the waste heat of the secondary effluent. Presently, the annual heat recovery from the heat pump system in Gaobeidian WWTP is only 69,610 MWh/y [33], less than 6% of the theoretical value. These results indicate that the residual energy from the secondary effluent has a great development value. Thus, a reasonable use of the low-grade energy contained in the effluent is also of great significance to the low-carbon operation of WWTPs.

Lastly, the annual heat recovery of the ventilation system was 20,238 MWh/y [33].

**Figure 2:**
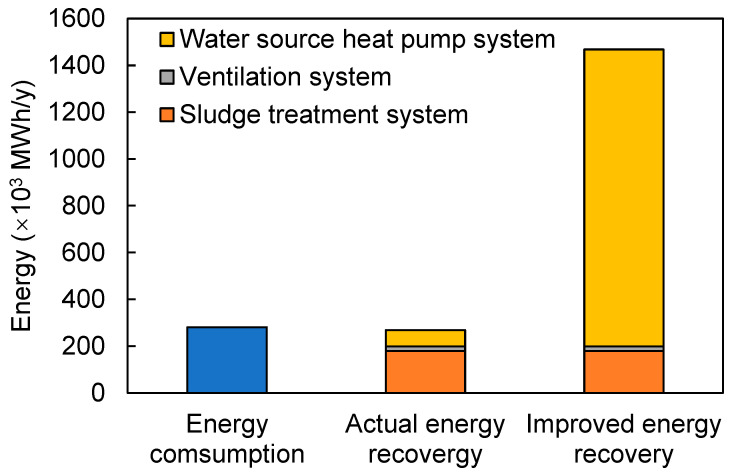
Annual energy consumption, actual energy recovery, and improved energy recovery after extracting heat from the secondary effluent in Gaobeidian WWTP.

In summary, the total energy recovery of Gaobeidian WWTP is 268,788 MWh/y (Figure 2), among which the energy recovery from the sludge treatment process (178,940 MWh/y) accounts for 66.6%, heat recovery from the secondary effluent (69,610 MWh/y) accounts for 25.9%, and heat recovery from the ventilation system (20,238 MWh/y) accounts for 7.5% (Table 4). Because the total energy consumption of the plant is 280,717 MWh/y, it has achieved 95.8% of energy neutralization. However, if the residual heat energy contained in the secondary effluent can be completely extracted, the total energy recovery would be increased to 1,468,515 MWh/y, which is 4.2 times greater than energy consumption. And the proportion of heat recovery from secondary effluent (1,269,337 MWh/y) rises to 86.4%, while those of energy recovery from sludge treatment process and heat recovery from the ventilation system decrease to 12.2% and 1.4%, respectively. The improvement of heat recovery technology can transform the WWTP into an energy generation factory.

### 3.2. The Carbon Footprint of Gaobeidian WWTP

#### 3.2.1. Direct GHG Emissions

This study considered only CO_2_ emissions from the external carbon source, but not CO_2_ emissions from the influent TOC conversion [34]. Gaobeidian WWTP uses sodium acetate as the external carbon source with an annual dosing of 10,950 t/y [7]. The corresponding direct CO_2_ emission from the oxidation of sodium acetate ($m_{CO_{2},\;\mathrm{carbon}\;\mathrm{source}}$) was 11,751 t/y. Furthermore, N_2_O emission from the wastewater treatment process ($m_{N_{2}O,\;\mathrm{st}}$) was calculated to be 507 t/y, while CH_4_ emission from the anaerobic stage of wastewater treatment ($m_{CH_{4},\;\mathrm{st}}$) was 2582 t/y.

The amount of CH_4_ released into the atmosphere during anaerobic digestion of sludge ($m_{ad,\;CH_{4}}$) was calculated using Equation (4) and estimated to be 543 t/y. Sludge of 1358 t/d with a water content of 80% entered the sludge treatment center for thermal hydrolysis and anaerobic digestion, which reduced the weight of sludge by 25%. As a result, the dry sludge used in land application (*W_land_*) was 74,351 t/y. The amount of N_2_O released from land application ($m_{land,\;N_{2}O}$) was 154 t/y, while that of CH_4_ ($m_{land,\;CH_{4}}$) was 223 t/y.

After converting all the amounts of GHGs into CO_2_ equivalents, the direct carbon emission from Gaobeidian WWTP was summarized and is shown in Table 5. Firstly, the direct carbon emission from the wastewater treatment process was 217,117 t/y. Although the amount of N_2_O emission during the wastewater treatment (507 t/y) was much lower than that of CO_2_ (11,751 t/y) and CH_4_ (2582 t/y), the carbon emission equivalent to N_2_O (151,136 t/y) was significantly higher than that of CO_2_ (11,751 t/y) and CH_4_ (54,230 t/y). Therefore, the carbon emission equivalent to N_2_O accounted for 69.6% of the direct carbon emissions during wastewater treatment. Secondly, the direct carbon emission from sludge treatment and disposal was 62,054 t/y, in which N_2_O contributed 74.1%. Therefore, the total direct carbon emission from Gaobeidian WWTP was 279,171 t/y. The direct carbon emission from the wastewater treatment process accounted for 77.8% and that from sludge treatment and disposal accounted for 22.2%. These results indicate that direct carbon emission from wastewater treatment is much higher than that from sludge treatment and disposal. In both processes, N_2_O emissions are a major contributor to direct carbon emissions.

#### 3.2.2. Indirect GHG Emissions

As described in 2.4, indirect GHG emissions include electricity, heat, and chemical consumption. Electricity was consumed for wastewater treatment (94,900 MWh/y), sludge treatment (20,795 MWh/y), pump station (52,080 MWh/y), administrative buildings (3793 MWh/y), and fuel transportation (96 MWh/y) (Table 2). As a result, the total electricity consumption of Gaobeidian WWTP was 171,664 MWh/y and the corresponding carbon emission from electricity consumption (*m_ec_*) was 103,685 t/y. Furthermore, thermal energy was used during the thermal hydrolysis of sludge (104,920 MWh/y) and consumed by the heating system of buildings (4133 MWh/y) (Table 2). The total heat consumption was 109,053 MWh/y, and its corresponding carbon emission was 43,185 t/y. The chemical consumption and its corresponding indirect carbon emission is shown in Table 6. The total indirect carbon emission from chemical consumptions was 20,427 t/y, with 75.0% coming from the use of sodium acetate. Therefore, the total indirect carbon emission from Gaobeidian WWTP was 167,297 t/y, with electricity, heat, and chemical consumption contributing to 62.0%, 25.8%, and 12.2% of the indirect carbon emission, respectively.

#### 3.2.3. Total GHG Emissions

**Figure 3:**
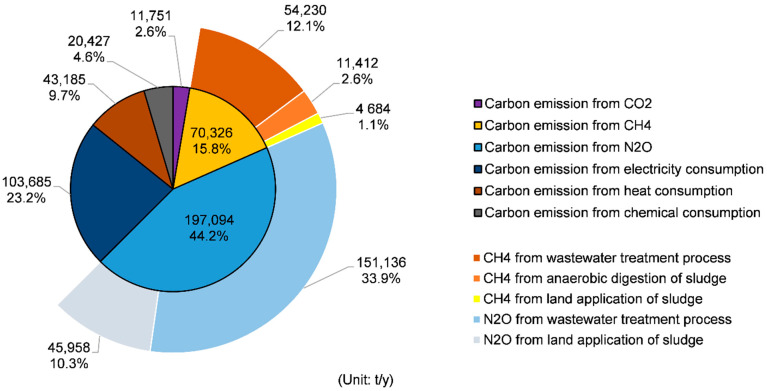
The composition of carbon emission from Gaobeidian WWTP.

In terms of the CO_2_ equivalent emission, the total annual carbon emission from Gaobeidian WWTP was 446,468 t/y, of which 279,171 t/y (62.5%) was the product of direct and 167,297 t/y (37.5%) was the product of indirect carbon emissions. Emissions from different processes are shown in Figure 3. Firstly, the direct carbon emission resulting from N_2_O emission (197,094 t/y) was the main contributor to GHG emissions, accounting for 44.2%. N_2_O was discharged from the wastewater treatment process (151,136 t/y equivalent carbon emission) and land application of sludge (45,958 t/y equivalent carbon emission), which accounted for 33.9% and 10.3% of the total GHG emissions, respectively. This demonstrates that efficient nitrogen removal technologies are urgently required to fully convert the substances containing nitrogen in sewage into nitrogen gas for the low-carbon operation of WWTPs. Then, the indirect carbon emission from electricity consumption was the second highest contributor (103,685 t/y, 23.2%) to the total GHG emissions, indicating that energy-efficient operation modes for WWTPs are needed, as well. The direct carbon emission resulting from CH_4_ emission (70,326 t/y equivalent carbon emission) was the third highest contributor to the total GHG emissions, accounting for 15.8%. Unexpectedly, CH_4_ emissions from wastewater treatment (54,230 t/y equivalent carbon emission, 12.1%) were much higher than those from anaerobic digestion (11,412 t/y, 2.6%) and land application of sludge (4684 t/y, 1.1%). This result could stem from the fact that most of the sewage treatment structures are open systems, so the CH_4_ directly enters the atmosphere. Although a large amount of CH_4_ was produced during anaerobic digestion, most of it was recovered for heat and power cogeneration. Therefore, biogas recovery is an efficient method to reduce CH_4_ emissions. Likewise, wastewater treatment technologies that lower CH_4_ emission are important in reducing GHG emissions. Following these contributors, indirect carbon emission from heat consumption, indirect carbon emission from chemical consumption, and direct CO_2_ emission accounted for 9.7%, 4.6%, and 2.6% of the total GHG emissions, respectively.

### 3.3. Carbon Emission Reduction

The carbon emission reduction resulting from water quality improvement (*J_wq_*) was calculated by comparing the GHG emission (CH_4_ and N_2_O) from wastewater after treatment with that from direct sewage discharge (Equation (10)), which was 113,878 t/y. The carbon emission reduction was 79,333 t/y when CH_4_ emission was reduced from wastewater, and it was 34,545 t/y when N_2_O emission was reduced. The former accounted for 69.7% of *J_wq_* while the later accounted for 30.3% of *J_wq_*.

As described in 3.1, the annual energy recovery capacity of the biogas system of the sludge treatment process was 178,940 MWh/y. The detailed composition of energy recovery from sludge treatment and the corresponding carbon emission reductions are listed in Table 7. The carbon emission reduction corresponding to energy recovery from sludge treatment was 79,813 t/y. In addition, an anaerobic ammonia oxidation system with a treatment scale of 3500 m^3^/d was used in treating sludge digestion liquid in the WWTP. Compared to previous techniques without the anaerobic ammonia oxidation system (ANAMMOX), the one with it save 3830 MWh/y of electrical energy, which corresponds to a carbon emission reduction of 2313 t/y [33]. The amount of dry sludge that can be used in land application (*W_land_*) was 204 t/d. As a result, the carbon emission reduction was 6932 t/y when sludge was used instead of commercial fertilizers (*S_land_*). Therefore, sludge treatment and disposal account for 89,058 t/y of total carbon emission reduction, of which energy recovery from the biogas system, electricity saving by treating sludge with ANAMMOX, and land application of sludge accounted for 89.6%, 2.6%, and 7.8%, respectively.

In addition, heat recovery from the ventilation system (20,238 MWh/y) corresponds to a carbon emission reduction of 8014 t/y.

According to the calculation of heat recovery capacity from the secondary effluent in Section 3.1, the annual electricity recovery from the heat pump system of the WWTP was 69,610 MWh/y, corresponding to a carbon emission reduction of 42,044 t/y. The theoretical energy recovery capacity from the secondary effluent is about 1,269,337 MWh/y, corresponding to a theoretical carbon emission reduction capacity of 766,680 t/y. This theoretical carbon emission reduction capacity is 17.2 times greater than the actual carbon emission reduction from secondary effluent. Therefore, there is still room for improvement when it comes to using residual thermal energy from the secondary effluent to reduce carbon emission.

All the mentioned carbon emission reductions are listed in Table 8. Based on the emission reductions, this study calculated the total carbon emission reduction of Gaobeidian WWTP. Currently, the total amount of carbon emission reduction is 252,994 t/y, which is mainly achieved through water quality improvement (45.0%), sludge treatment and disposal (35.2%), residual thermal energy recovery from the secondary effluent (16.6%), and heat recovery from the ventilation system (3.2%).

### 3.4. Carbon Neutralization Evaluation

**Figure 4:**
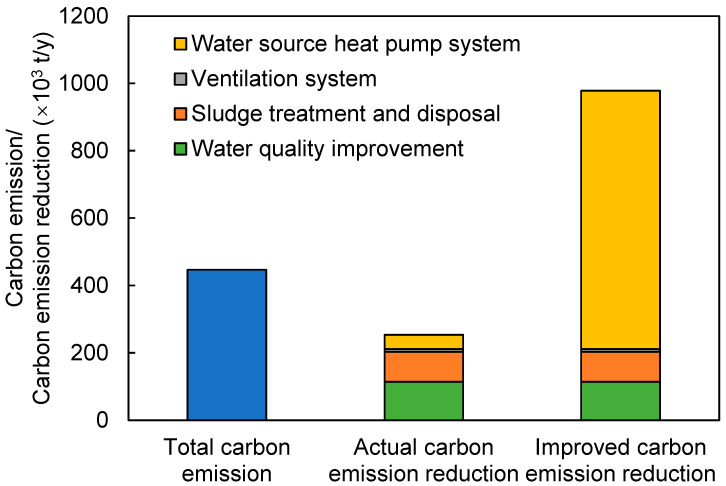
Carbon emission, actual carbon emission reduction, and improved carbon emission reduction after the complete extraction of heat from the secondary effluent in Gaobeidian WWTP.

The amounts of carbon emitted from and reduced by Gaobeidian WWTP are summarized and compared in Figure 4. The current amount of carbon emission reduction (252,994 t/y) is still much lower than emitted (446,468 t/y), with a carbon neutralization ratio of only 56.7%. This indicates that the operation of Gaobeidian WWTP is far from carbon-neutral, although it already achieved near energy-neutral operation (95.8% energy neutralization as described in Section 3.1).

If the residual thermal energy from the secondary effluent is fully developed and recovered, the plant is expected to achieve around 977,630 t/y of total carbon emission reduction. The proportion of carbon emission reduction as a result of heat recovery from secondary effluent increases to 78.4%, while that resulting from water quality improvement, sludge treatment and disposal, and heat recovery from the ventilation system decreases to 11.6%, 9.1%, and 0.8%, respectively. That means that heat recovery from the secondary effluent will be the main contributor to carbon emission reduction instead of water quality improvement. The carbon neutralization ratio after waste heat recovery can be as high as 219.0% (Table 9), indicating that Gaobeidian WWTP has the potential to transform from a carbon emission plant to a carbon sink.

The current amount of carbon emission from Gaobeidian WWTP shows that it is difficult to reach carbon neutrality by solely relying on the wastewater treatment process and biogas cogeneration. Carbon neutrality is not equivalent to energy neutrality, although energy neutrality has almost been achieved in the plant. Improving the efficiency of heat pump stations to recover thermal energy from the secondary effluent is the key to achieving carbon neutrality.

### 3.5. Measures to Reduce the Carbon Footprint

Two paths can be taken to achieve low-carbon operation of WWTPs. One relies on increasing carbon sinks as many as possible by optimizing the sludge digestion process to improve energy recovery from sludge, devoting attention to the residual thermal energy in the secondary effluent, and using self-generated clean energy. The other path relies on reducing the amount of carbon emission during the operation of the plant by upgrading the aeration system, optimizing the chemical dosing section, improving the pipe network system, and renovating equipment for lower carbon emission. The actions of government departments and the development of an ecological civilization are also important for achieving carbon neutrality in WWTPs. WWTPs should continue to explore energy-saving modes and systematically plan environmental water management so as to utilize resources under the premise of eco-friendliness.

## 4. Conclusions

The current energy consumption of Gaobeidian WWTP is 280,717 MWh/y, and its energy recovery is 268,788 MWh/y, resulting in an energy neutralization ratio of 95.8%. Therefore, from the perspective of energy balance, energy neutrality has almost been achieved in the plant. However, from the perspective of carbon footprint, the current amount of carbon emission from the WWTP is 446,468 t/y, and the amount of carbon emission reduction is 252,994 t/y, resulting in a carbon neutrality ratio of only 56.7%. These indicate that although the plant almost reached energy neutrality, it still has a long way to go before reaching carbon neutrality. In order to increase the amount of carbon emission reduction, a sustainable and feasible method of recovering waste heat from secondary effluent was proposed. If the waste heat of the secondary effluent can be completely extracted, the total energy recovery of the plant would be increased to 1,468,515 MWh/y, and the carbon emission reduction would be increased to 977,630 t/y. In this context, the energy and carbon neutralization ratio could be as high as 523.1% and 219.0%, respectively. That means that the WWTP can become a power production unit and a carbon sink.

## Figures and Tables

**Table 1:** Qualities of the influent and effluent in Gaobeidian WWTP.

Water Quality Indexes	Influent (mg/L) [24]	Effluent (mg/L) [24]	Removal Efficiency (%)	Effluent Standards (mg/L) [23]
COD	283	15	95%	30
BOD_5_	175	2.5	99%	6
TP	6.90	0.059	99%	0.3
TN	50.6	10.9	78%	15
SS	239	<5	>98%	5
Chroma	50	5.4	89%	15
NH_4_^+^-N	44.28	0.45	99%	1.5

**Table 2:** Energy consumption of Gaobeidian WWTP [28].

Projects	Energy Consumption (MWh/y)
Electricity consumption of the wastewater treatment process	94,900
Electricity consumption of the sludge treatment unit	20,795
Thermal hydrolysis of sludge	104,920
Electricity consumption of the pump station	52,080
Heating system for buildings	4133
Electricity consumption of administrative buildings	3793
Electricity consumption of fuel transportation	96
**Total**	280,717

**Table 3:** Theoretical heating and cooling capacity of the water-source heat pump system in Gaobeidian WWTP.

Project	COP [32]	Heating/Cooling Capacity (GJ/y)	Equivalent Electricity (MWh/y)	Energy Consumption by Water-Source Heat Pump (MWh/y)	Net Energy Production (MWh/y)
Heating capacity	4.24	7,417,487	2,060,413	485,947	1,574,467
Cooling capacity	4.16	4,569,615	1,269,337	305,129	964,208

**Table 4:** Calculations of energy recovery in Gaobeidian WWTP.

Project	Actual Energy Recovery (MWh/y)	Proportion (%)	Energy Recovery after Residual Thermal Energy Utilization (MWh/y)	Proportion (%)
Biogas system	178,940	66.6	178,940	12.2
Ventilation system	20,238	7.5	20,238	1.4
Water source heat pump system	69,610	25.9	1,269,337	86.4
**Total**	268,788	100	1,468,515	100

**Table 5:** Direct carbon emissions from Gaobeidian WWTP.

Emission Process	GHG Species	GHG Emission (t/y)	Carbon Emission (t/y)	
Wastewater treatment	CO_2_	11,751	11,751	217,117
	CH_4_	2582	54,230	
	N_2_O	507	151,136	
Sludge treatment and disposal	CH_4_ (from anaerobic digestion)	543	11,412	62,054
	CH_4_ (from land application)	223	4684	
	N_2_O (from land application)	154	45,958	
**Total**				279,171

**Table 6:** Chemical consumption and its indirect carbon emission.

Chemicals	Annual Consumption (t/y) [28]	Usage	Carbon Emission Factor (kg CO_2_/kg)	Indirect Carbon Emission (t/y)
Sodium acetate	10,950	External carbon source	1.4	15,330
Sodium hypochlorite	4562.5	Disinfectant	0.89	4061
Polyacrylamide (PAM)	461.7	Dewatering flocculant	1.9	877
Fe_2_O_3_	25.5	Dry desulfurizer for biogas	1.3	33
FeCl_3_ (38%)	601.4	Desulfurizer in digester	0.18	108
Polyaluminum chloride (PAC)	32.9	Chemical phosphors removal	0.537	18
**Total**				20,427

**Table 7:** Energy recovery from sludge treatment and its corresponding carbon emission reduction.

Total Energy Recovery from Sludge Treatment Process (MWh/y)	Energy Recovery Pathway	Energy (MWh/y) [12]	CO_2_ Emission Factor of Electricity/Heat	Carbon Emission Reduction (t/y)
178,940	Electrical energy from heat and power cogeneration	43,040	0.604 t/MWh	25,996
	Heat recovery from flue gases	19,370	0.11 t/GJ	7671
	Heat recovery from water cylinder jackets	24,750		9801
	Heat recovery from boiler steams	91,780		36,345
**Total**				79,813

**Table 8:** Calculation of the total carbon emission reduction.

Carbon Emission Project		Present Carbon Emission Reduction (t/y)	Proportion (%)	Carbon Emission Reduction after Residual Thermal Energy Utilization (t/y)	Proportion (%)
Water quality improvement		113,878	45.0	113,878	11.6
Sludge treatment and disposal processes	Biogas system	79,813	31.5	79,813	8.2
	Sludge land application	6932	2.7	6932	0.7
	Anammox system	2313	0.9	2313	0.2
Ventilation system		8014	3.2	8014	0.8
Water source heat pump system		42,044	16.6	766,680	78.4
**Total**		252,994	100	977,630	100

**Table 9:** Carbon footprint calculations.

Type	Carbon Emission (t/y)	Carbon Emission Reduction (t/y)	Carbon Sink (t/y)	Carbon Neutrality Rate (%)
Actual	446,468	−252,994	193,474	56.7%
After residual thermal energy utilization	446,468	−977,630	−531,162	219.0%

## Data Availability

The data presented in this study are available in article here.
